# Cardiac metastasis from a renal cell carcinoma

**DOI:** 10.11604/pamj.2015.22.167.7130

**Published:** 2015-10-21

**Authors:** Zairi Ihsen, Mzoughi Khadija, Jnifene Zouhayer, Fennira Sana, Ben Moussa Fethia, Kammoun Sofiene, Kraiem Sondos

**Affiliations:** 1 Department of Cardiology, Habib Thameur Public Hospital, Bab El Fallah, Tunis, Tunisia

**Keywords:** Renal cell carcinoma, cardiac metastasis, myocardium, cardiac tumors

## Abstract

Here we report a case of asymptomatic right ventricular tumor, for which surgical removal was done. Pathology reveals that the mass is a metastasis of a renal carcinoma.

## Introduction

Metastatic disease of the heart is over twenty times more common than primary heart tumors [1]. They are among the least known and highly debated issues in oncology, and few systematic studies are devoted to this topic. Cardiac involvement in renal cell carcinoma (RCC) commonly arises from direct tumor thrombus extension into the inferior vena cava. The second mechanism of metastasis involves tumor dissemination via hematogenous spread [2]. Cardiac metastasis in the absence of vena cava extension is exceedingly rare, with only a few cases reported in the literature. In this report, we describe the case of ventricular metastases from RCC in the absence of vena cava involvement.

## Patient and observation

**Figure 1:**
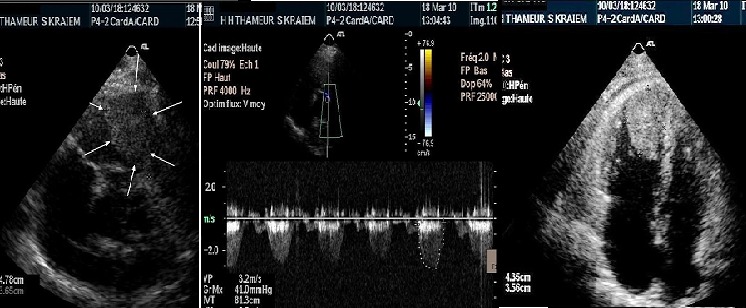
Echocardiography showing the right ventricular mesuring 43 cm long axis with important pulmonary artery obstruction assessed by the pulmonary mean gradient

**Figure 2:**
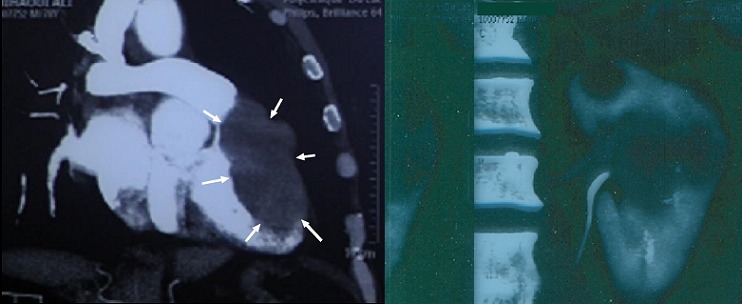
CT scan showing renal tumor and heart tumor

**Figure 3:**
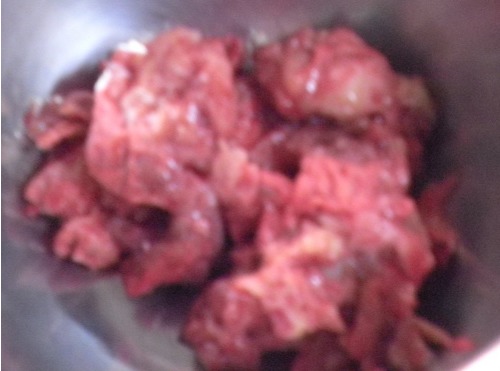
Photograph of the explanted tumor

An 81-year-old woman was admitted to Habib Thameur Hospital because of progressive dyspnea and atypical chest pain. No past medical history of cardiovascular risk factors. Clinical examination found no signs of congestion. Cardiac auscultation revealed normal heart sounds and a systolic murmurin pulmonary valve area. Trans thoracic echocardiogram exam demonstrated a right ventricular mass that measures 43 cm of diameter probing into the pulmonary artery with an intermittent pulmonary artery obstruction (Figure 1). Right chambers were dilated with important pulmonary arterial hypertension. CT scan reveals the right ventricular tumor and multiple left renal tumors with an extension to the left suprarenal glands and the the pelvicaly ceal system. No inferiorvena cava involvement was detected (Figure 2). Cardiac tumor resection surgery was indicated. The tumor was resected and addressed for pathology exam that revealed Clear cell tubule papillary renal cell carcinoma (Figure 3). The post operative course was uneventful and the patient was transferred to the urology department few days later. This patient was viewed five months later and she was totally asymptomatic.

## Discussion

Cardiac metastases are 20 to 40 times more common than primary cardiac malignancies, and have been reported in different studies in approximately 2% to 18% of cases at autopsies. Intra myocardial metastases arise most often in the course of malignant melanoma, leukemia, lymphoma, and lung, esophageal and breast cancers [3]. The tumours showing the highest rate of heart metastasis were the following: pleural mesothelioma (48.4%), melanoma (27.8%), lung adenocarcinoma (21%), undifferentiated carcinomas (19.5%), lung squamous cell carcinoma (18.2%) and breast carcinoma (15.5%). High rates of heart metastatisation have also been observed in patients affected by ovarian carcinoma (10.3%), lymphomyelo proliferative neoplasms (9.4%), bronchioalveolar carcinomas (9.8%), gastric carcinomas (8%), renal carcinomas (7.3%) and pancreatic carcinomas (6.4%) [4]. Approximately 45% of patients with renal carcinoma present with localized tumors, 25% of patients present with locally advanced disease, and approximately 30% of patients may have metastases at the time of diagnosis. The most common metastatic sites are the lungs, bone, soft tissues, liver and central nervous system. Heart involvement via the inferior vena cava (IVC) is a well-known phenomenon in clear cell renal cell carcinoma cases. Renal cell carcinoma is known for invading the renal vein and further promoting tumor thrombosis of the vena cava and even the right atrium [5].

About two thirds of all cardiac metastases involved the pericardium (69.4%), one-third the epicardium (34.2%) or the myocardium (31.8%) and only 5% the endocardium. Endocardial metastases, usually localized to the right heart, was especially associated with renal carcinoma [4]. Clinical presentations of cardiac metastasis are highly variable and dependent on most involved site. While they are commonly asymptomatic, hypertension is the most common cardiac presentation of renal cell carcinoma and occurs in 20% to 37.5% of patients. Other cardiac presentations include shortness of breath, cough, arrhythmia, chest pain and peripheral edema [1]. Syncope is most often due to vasovagal reactions. Other causes include tachydys rhythmias, heart block, obstruction ofleft ventricular out flow and orthostatic hypotension. Surgical resection can play an important role in the palliation of isolated cardiac metastasis [1]. Unlike most other neoplasms, renal cell carcinoma is relatively resistant to classic chemotherapeutic agents, but has demonstrated some sensitivity to immunotherapy [6].

## Conclusion

Cardiac metastasis are not rare, even more frequent than primary heart tumors; ultra-sound examination of the heart should be performed as soon as symptoms develop.
